# The Configuration of Incentives in Small and Medium-Sized Content Platform Enterprises Under the Normalization of COVID-19

**DOI:** 10.3389/fpubh.2022.885729

**Published:** 2022-04-29

**Authors:** Yingying Zhou, Jianbin Chen, Baodong Cheng

**Affiliations:** ^1^School of Economics and Management, Beijing Forestry University, Beijing, China; ^2^School of Business, Beijing Union University, Beijing, China

**Keywords:** social incentives, market-oriented incentives, knowledge collaborative performance, COVID-19, fuzzy set qualitative comparative analysis

## Abstract

The COVID-19 pandemic has made the advantages of online knowledge communities with cross-space, time, interpersonal, and other characteristics fully demonstrated. Exploring the configurations of platform incentives to improve knowledge collaboration performance can provide a reference for the efficient and sustainable development of the platforms under the normalization of the COVID-19 pandemic. With the help of the fuzzy set qualitative comparative analysis method, taking the social Q&A platform enterprises as an example, this study analyzed the configurations of platform incentives for the high knowledge collaborative performance from the dimensions of market incentives and social incentives, and the heterogeneity of knowledge capital appreciation and social capital appreciation is also discussed. The results show that each of the platform incentives (virtual currency, cash benefit, community reputation, social identity, skill training, and communication) does not constitute a necessary condition for high performance. There are four and three types of configurations for intellectual capital appreciation and social capital appreciation, respectively. The important findings are that nearly 30% of users may participate in knowledge production based on pure economic benefits, and there may be so-called “get the best deal” behavior. Social identity, community reputation, and skill training have an important incentive effect on knowledge collaboration. Communication supplemented by a little economic incentives can significantly promote the appreciation of social capital.

## Introduction

The COVID-19 pandemic has severely disrupted the functioning of global postsecondary institutions since 2020 (1). The massive and uncontrolled spread of the COVID-19 virus in various parts of the world has made the WHO officially announce that COVID-19 has been a world pandemic since March 11, 2020. The pandemic was announced not only as a medical need preparedness, but preparedness for various aspects will be affected, both in the social, cultural, and economic fields (2). Small and medium-sized enterprises are the main force of national economic and social development, which are important to stabilizing economic growth, enhancing economic activity, ensuring the integrity of the production system, and stabilizing employment (3). Due to the low resilience, most small and medium-sized enterprises are more seriously affected by the COVID-19 pandemic, while the content platform enterprises are the opposite. In the Internet age, the “Online Knowledge Community” (OKC) which integrates the functions of “knowledge sharing” and “online social networking,” emerged as the times require (4). During the epidemic, OKC has provided a convenient way for the public to interact with knowledge and socialize with its characteristics of spanning time, space, interpersonal, and other constraints, and it has also highlighted the value and advantages of OKC. However, in the post-epidemic era, although OKC faces great opportunities, the huge information capacity, the random insertion, and editing at any time have greatly increased the amount of information and the degree of confusion, the low willingness to participate in knowledge collaboration is also the challenge that the OKC faces.

First of all, the traditional mechanism of knowledge collaboration in OKC is based on social User Generated Content (UGC) (5). Second, as the users' scale increases, market-oriented mechanisms and bureaucratic management gradually emerge (6). In particular, the commercialization of OKC has entered a new stage after the introduction of means such as signing contracts and cash rewards (7). Third, the excessive use of market incentives will lead to the loss of knowledge quality and social interaction (8, 9). Monetary incentives may cause the Matthew effect (A small number of experts contribute a lot of answers and profits.) and speculation (Ask a lot of questions to earn money, or collude between questioner and answers to create a hot illusion.). The payment mechanism suppresses the update frequency of topics and the number of user comments and answers, and it does not generate more content or attract more users to participate, while it changes the interaction and collaboration between users. OKC knowledge collaboration arises from niche social production and interpersonal trust, so, how to balance social incentives and market-oriented incentives, interpersonal trust and system trust for sustainable development in the pursuit of user scale and commercial interests, has become a new challenge. In this context, exploring the knowledge collaboration mechanism and the corresponding platform incentives in OKCs has attracted the attention of many scholars. With the increase in people's demand for remote knowledge interaction and social interaction caused by the epidemic, it is of great practical and theoretical significance to explore the platform incentives for content platform companies to improve users' knowledge contribution level and performance.

Economists often emphasize that “incentives matter.” The basic “law of behavior” is that higher incentives will lead to more effort and higher performance. Research on OKC's knowledge collaboration mechanism shows that obtaining economic returns is only one of the many motivations for users to participate in knowledge collaboration (10). OKC introduces economic incentives based on the assumption of “economic man” to enhance the active participation of contributors (7). However, the quality of contributed knowledge will not improve as a result (8), the more you pay, the answers you get will be longer, more, and richer, while it does not mean that the questioner will get better answers (9, 11). And to a certain extent, economic incentives have erosive and spillover effects on UGC creation, and the combination of target incentives and challenge incentives can effectively prevent this erosive effect (7). This also means that knowledge production that mainly relies on social benefits may face the problem of insufficient contribution. OKCs have obvious characteristics of user self-organization and participation (12), and interests are the main driving factors for users to participate in OKC knowledge sharing. Only by combining economic returns with social returns (such as consolidating users' interests and hobbies) can we truly grasp the transformation of user identity and encourage users to participate (12).

Based on the above analysis, this article attempts to answer the following questions: Are and to what extent certain incentives are necessary for high knowledge collaboration performance (KCP)? How are these elements coupled to achieve high KCP? Is there any heterogeneity in the influence of different incentives and their configurations on the appreciation of intellectual capital and social capital?

**Figure 1:**
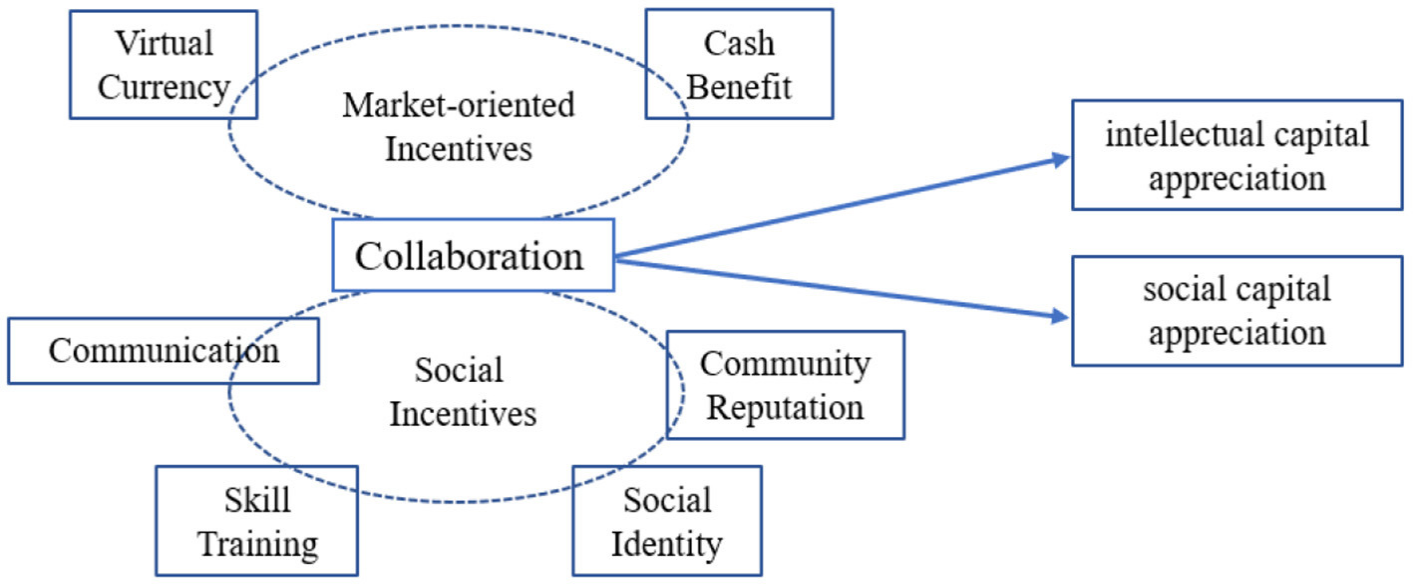
Research model.

The possible marginal contribution of this article is that, first, it analyzes the impact of platform incentives on KCP from the two dimensions of intellectual capital appreciation (ICA) and social capital appreciation (SCA). Second, it systematically analyzes the configurations of platform incentives to improve KCP from the aspects of market-oriented incentives and social incentives. The research conclusions are beneficial for the users and platforms. On the one hand, for the users, different incentives on the platform can meet the heterogeneous needs of different users, thereby improving their knowledge collaboration performance. On the other hand, the success of a content platform enterprise depends largely on user satisfaction and other factors that eventually increase users' intentions to continue participating, therefore, the conclusions provide a reference for content platform companies to better manage and motivate users in the post-COVID-19 era, and to build a sustainable platform ecology. The research model of this study is shown in Figure 1.

## Literature Review

### Platform Incentives

A major challenge in knowledge management is motivating people to share their knowledge with others (10, 13). In many OKCs, this challenge is addressed through an incentive system associated with users' knowledge-sharing activities. As one of the main influencing factors of OKC knowledge collaboration, platform incentives have attracted the attention of many scholars. At the theoretical level, a social cognitive theory is the most specific in explaining how different types of incentives affect performance (10). Regarding the platform incentive mechanism, Ryan and Deci (14) proposed that perceived usefulness and perceived ease in the process of user knowledge creation are the internal and external motivations for UGC. Among them, external motivation is based on the exchange of tangible or intangible assets, including non-material external benefits, tangible rewards, and mutual benefit expert training incentives, which will prompt users to engage in content creation. Intrinsic motivation is manifested in the positive promotion of self-efficacy, hedonic experience, social needs, and values to UGC (15). Smith et al. (16) believe that the motivation for content creation is from high to low: entertainment, gaining a sense of identity, recording and self-expression, acquiring social capital, enhancing social interaction, and obtaining rewards. It can be seen that from the perspective of incentive forms, existing research generally divides OKC platform incentives into material incentives (such as prize, monetary incentives, and point incentives), spiritual incentives (such as grade medals, rankings, identity tags, task-driven, and privilege incentives), and competency incentives (such as training and communication).

Material incentives are a direct means of giving feedback to users' knowledge contribution behaviors in a market-oriented way. This article calls them market-oriented incentives, which mainly include virtual currency and cash rewards (17). Unlike the incentives imposed by many systems, market-oriented incentives support the direct exchange of monetary benefits between individuals, creating an interesting gift economy model and enabling widespread “user generation” (15). As the most commonly used means of market incentives (10), virtual currency and cash rewards are also commonly used in OKC to improve user participation. According to the classic “economic man” hypothesis, the economic incentives in OKC can encourage people to create knowledge (7), because we are always willing to act to increase our interests, especially monetary ones (18). However, there are also a large number of studies from psychology and economics showing that economic incentives do not always perform well (19), and they will erode people's existing intrinsic motivation and bring about negative effects in some situations (7).

Non-monetary incentives, especially spiritual incentives such as social identity, can also produce incentive effects derived from current and future positive reinforcement, and play an important role in generating knowledge activities (20). Relevant studies have shown that obtaining economic rewards is only one of the motivations for users to participate in knowledge collaboration, and users' knowledge sharing behavior in OKC is more about seeking spiritual recognition and satisfaction (10). Although anonymity is the general “rule” of online communication, members often seek to create “online identities” to identify each other and may engage in various levels of self-disclosure (21), once social proof is established, members become psychologically connected to the group and experience its successes and failures, so that they are willing to support the group with which they identify and take pride in its activities. Community reputation is the feeling of earning respect or improving status by contributing knowledge in OKC (22, 23), representing superior ability and high strength, and can also bring a sense of achievement to users (22), therefore, building reputation is a powerful motivator for knowledge sharing (22).

In addition to material and spiritual benefits, obtaining useful information and skills, that is, skill training is also the most direct motivation for users, especially knowledge seekers, to participate in knowledge collaboration in OKC (12). This intrinsic motivation is more autonomously oriented and results from a person's intrinsic interest or joy in the activity (e.g., doing something because it is fun) (24). Communication is the basis of knowledge collaboration in OKC, including knowledge sharing, transformation, and integration (25). When users participate in knowledge interaction and generate new perspectives on related issues, this interaction will positively promote the knowledge collaboration process (26). There are also studies suggesting that communication can change the attitude of members, thereby changing the degree of identification with the organization. Supportive communication through shared understanding and organizational identity has a positive impact on knowledge sharing (26).

To sum up, this research analyzes the aspect of market-oriented incentives and social incentives and believes that market-oriented incentives include virtual currency and cash benefits. And social incentives include social identity, community reputation, skills training, and communication.

### Knowledge Collaboration Performance

Knowledge collaboration performance (KCP) in OKC is the ultimate value realization method of knowledge. At present, there is no unified definition for it in the academic world (4), while the conclusion that it includes the dimensions of ICA and SCA has been recognized by many scholars (19, 27, 28). Social capital in a virtual community represents the connection between people and the personal wealth accumulated through the connection, which is the trust cooperation and collective behavior established in the interpersonal network of the community (29). The social capital theory believes that the network of relationships embodied by individuals has an impact on interpersonal knowledge-sharing behavior (30). In its simplest form, social capital is what an individual knows about someone that extends what you have (economic capital) or know (human capital). A basic assumption about social capital is that social systems have immediate or expected value (31). The success of viral marketing, open-source communities, and social media makes the purpose of social capital very attractive (32). Therefore, SCA has also become one of the main purposes for users to participate in knowledge collaboration in OKC. Social capital includes three dimensions: structural dimension, relational dimension, and cognitive dimension (28). The structural dimension measures the social connection status, that is, the relationship existing among members; the relationship dimension emphasizes the strength of the relationship, which is reflected in the individual's sense of trust, recognition, and reciprocity for other users in OKC, that is, when an individual gets help from others, he will give each other in return (33). The social capital of the cognitive dimension is mainly reflected in the shared vision of OKC members, such as common interests, opinions, and values (34).

Compared with SCA, ICA is more direct (35), which is reflected in the acquisition of user knowledge. Due to the sharing and non-attrition of intellectual capital, the appreciation of intellectual capital is not only manifested in the increase of explicit knowledge (experience summary, process documentation, knowledge base, and so on), or the final explicit knowledge product delivered to customers, the implicit knowledge achievement is also the value-added part of knowledge capital, which is manifested as the improvement of individual and team ability, accumulation of experience, and improvement of the process (35). The explicit ICA mainly measures the knowledge achievements ultimately formed by knowledge collaboration and jointly owned by organizations or teams, such as patents, processes, and regulations. The tacit ICA mainly measures the increase of individual experience and skills, the improvement of team ability, organizational culture, and practice (36).

## Materials and Methods

### Questionnaire Design and Data Collection

Our research subjects are all from China, and they are all users of one or more social Q&A content platforms, including Zhihu, Baidu zhidao, Douban, Yahoo!Answers, Sogou wenwen, and other professional forums. First, we conducted systematic research and discussion on the platforms, and then, we designed the questionnaire concerning the existing research and invited relevant experts to modify it. In addition, before the start of the formal survey, we conducted a preliminary survey, combined with the interviews with users, and further revised the questionnaire. Finally, we collect data with the help of a professional research company. This kind of survey process provides greater control and is getting embraced by researchers (27).

The survey was conducted in August 2021 and a total of 210 questionnaires were returned, including 201 valid questionnaires, with an effective response rate of 95.71%. Table 1 gives the basic sample characteristics.

**Table 1:** The basic characteristics of respondents.

**Characteristics**	**No. of responses**	**(%)**	**Characteristics**	**No. of responses**	**(%)**
Gender			Usage behavior		
Male	107	53.23%	Browse knowledge	175	87.06%
Female	94	46.77%	Search knowledge	163	81.09%
Age			Post a question	111	55.22%
Under 20	1	0.00%	Upload files (text, picture. etc.)	47	23.38%
20–30	62	22.39%	Personal creation	38	18.91%
30–40	109	21.39%	Answer the questions	101	50.25%
40–50	25	29.85%	Post or repost	96	47.76%
Over 50	4	26.37%	Comment or like	148	73.63%
Use days per month		Usage time			
<5 days	0	0.00%	<1 year	3	1.49%
5–10 days	45	22.39%	1–3 years	43	21.39%
10–15 days	43	21.39%	3–5 years	91	45.27%
15–20 days	60	29.85%	5–7 years	47	23.38%
More than 20 days	53	26.37%	More than 7 years	17	8.46%

### Measurements

All data-related questions included a seven-point scale from one (not important) to seven (extremely important). The questions were divided into three sections: users' basic information, platform incentives (social incentives, market-oriented incentives), and KCP. To assure validation of the instrument, survey items were mostly adapted from scales developed and validated by previous studies. Among them, market-oriented incentives refer to the research of Jin (37) and Zhao (34). Social incentives refer to the research of Lucas and Ogilvie (38), Rafaeli et al. (20), Kumi and Sabherwal (39), Bai et al. (6), and Nan (40). And KCP refers to the research of Chang and Chuang (29), Chow and Chan (33), Chen et al. (35), and Zhou et al. (4).

### Method

We used fuzzy set qualitative comparative analysis (fsQCA) to analyze the configurations of platform incentives leading to high KCP. The QCA was developed by Ragin (41, 42) to analyze complex causality through the identification of the sufficient and necessary conditions for the occurrence of a phenomenon based on Boolean algebra and set theory (43). And it can provide a more refined analysis of complex management issues such as heterogeneity between cases, concurrency conditions, asymmetric relationships, and equivalence paths (42). In particular, QCA provides configurations of conditions that emerge from its algorithm. Configurations can be seen as outcome variables, and conditions somewhat resemble explanatory variables. The key difference between QCA and other symmetric methods is that QCA allows for conditions to be part of several configurations, that is, outcomes. In other words, while symmetric methods allow variables to have only a one-sided effect, QCA removes that restriction (44). QCA has three main variations according to variable type: crisp set QCA (csQCA), multi-value QCA (mvQCA), and fuzzy-set QCA (fsQCA). This article selects the widely used fsQCA to analyze the configuration paths of platform incentives leading to high KCP.

The fsQCA allows researchers to deal with conjunctural causality. The fsQCA identifies multiple causal recipes between different initial conditions to the same final state. In addition, large samples are not mandatory to use the fsQCA (42, 45). The fsQCA requires the calibration of partial memberships in the sets (42, 46). This calibration divides membership into meaningful groupings by using values between zero (non-membership) and one (full membership) (41). That means we distinguish cases that are either fully in, fully out, or in between certain sets (44, 47). Our data show conditions that can take intermediate values. Hence, we divide our values into 5 percentiles according to the research by Pappas and Woodside (48). And the three thresholds of each variable are shown in Table 2.

**Table 2:** The calibration criteria for the variables.

**Conditions tested**		**Full member**	**Cross-over**	**Non-member**
Market-oriented Incentives	Virtual currency	6.6667	5.3333	2.6667
	Cash benefit	6.6667	5.3333	1.6667
Social Incentives	Social identity	6.6	5.6	3.4
	Community reputation	6.75	5.75	3
	Skill training	7	6	4
	Communication	6.75	5.75	3.75
Knowledge collaboration performance	ICA	6.5	5.8333	3.5
	SCA	6.5	5.6667	4.2

## Results

### Analysis of the Necessary Conditions

The fsQCA starts with the conditions necessary for the outcome (42, 49). The causal condition's degree of necessity indicates the degree to which that condition is necessary to achieve the outcomes (47). For a condition to be necessary, it should present a consistency score that exceeds the threshold of 0.90 (42). Taking ICA and SCA as the outcome variables, respectively, the necessity of each antecedent condition is analyzed, and the results are shown in Table 3.

**Table 3:** Results of the analysis of the necessary conditions.

**Antecedent variables**	**ICA**		**SCA**	
	**Consistency**	**Coverage**	**Consistency**	**Coverage**
Virtual currency	0.745490	0.856598	0.850306	0.801012
~ Virtual currency	0.470520	0.662452	0.485445	0.560332
Cash benefit	0.683636	0.845816	0.785189	0.796441
~ Cash benefit	0.544405	0.704910	0.562090	0.596687
Social identity	0.759290	0.909253	0.832887	0.817696
~ Social identity	0.484870	0.650405	0.515245	0.566632
Community reputation	0.729119	0.885936	0.813148	0.810032
~Community reputation	0.502697	0.663567	0.527427	0.570781
Skill training	0.739199	0.944270	0.869527	0.869794
~ Skill training	0.508972	0.638021	0.489061	0.526893
Communication	0.767343	0.936255	0.822423	0.861309
~ Communication	0.468876	0.616153	0.535886	0.550735

From Table 3, we can see that all platform incentives leading to high performance exceed the consistency threshold of 0.75, indicating that all platform incentives are sufficient for high performance. Since no condition exceeds 0.90, the platform incentives are not necessary conditions for high performance (47), which occurs with the negation of all conditions.

### The Configurations of Platform Incentives Leading to High KCP

The data analysis continues with the construction of a truth table to identify the configurations of conditions that are related to KCP. Following best practice, we build truth tables based on the standard of consistence = 0.8 and number = 2 (48), and we report the core and peripheral conditions: core conditions are in parsimonious and intermediate solutions, while peripheral conditions are only part of intermediate solutions (42, 47). We report the causal configurations solutions that lead to ICA in Table 4 and SCA in Table 5.

**Table 4:** The configurations leading to high ICA.

**Conditions**	**Configurations**									
	**1**	**2a**	**2b**	**2c**	**2d**	**3a**	**3b**	**4a**	**4b**	**4c**
Virtual currency	*			⊗	⊗	∙		*	*	*
Cash benefit	*	⊗	⊗	⊗	⊗		∙	*	*	*
Social identity	⊗		*		⊗	*	*	*		*
Community reputation		⊗	⊗	*		*	*		*	*
Skill training	⊗	*	⊗	⊗	*	*	*			
Communication		*	⊗	⊗	⊗			*	*	
Consistency	0.8680	0.9741	0.9013	0.8804	0.9336	0.9726	0.9638	0.9606	0.9597	0.9441
Raw coverage	0.2986	0.3075	0.2633	0.2534	0.2636	0.5459	0.5216	0.5386	0.5342	0.5447
Unique coverage	0.0049	0.0171	0.0053	0.0056	0.0151	0.0366	0.0120	0.0157	0.0095	0.0044
Overall solution consistence	0.8856									
Overall solution coverage	0.7592									

Black circles (∙) indicate the presence of a condition, and circles with x (⊗) indicate its absence. Circles with * mean core condition and circles without * mean peripheral condition. Black spaces mean “does not care” condition.

**Table 5:** Causal configurations leading to SCA.

**Conditions**	**Configurations**						
	**1a**	**1b**	**2a**	**2b**	**3a**	**3b**	**3c**
Virtual currency		∙	∙		*	*	*
Cash benefit	⊗		*	*	∙	∙	
Social identity		⊗	*	*			*
Community reputation	⊗	⊗		*	*	*	*
Skill training	∙	⊗		*		⊗	*
Communication	*	*	*		*		
Consistency	0.9481	0.9337	0.9481	0.9388	0.9405	0.8922	0.9422
Raw coverage	0.365	0.3178	0.6485	0.6197	0.6386	0.4249	0.6450
Unique coverage	0.0381	0.0027	0.0204	0.0158	0.0081	0.0136	0.0433
Overall solution consistence	0.8839						
Overall solution coverage	0.8240						

Black circles (∙) indicate the presence of a condition, and circles with x (⊗) indicate its absence. Circles with * mean core condition and circles without * mean peripheral condition. Black spaces mean “does not care” condition.

The results show that (see Table 4) there are four kinds of configuration paths for ICA. The overall coverage is 0.7592, which indicates that the combined solutions account for ~75.92% of the membership. And all the configurations regarding the presence of the ICA in this study present consistency levels above the 0.80 threshold suggested by Ragin (42) and Fiss (47), indicating that the four types of configurations have good explanatory power for the results. The raw coverage of third and fourth configurations is higher, indicating that these types of incentive configurations are more likely to lead to ICA.

Table 5 shows three types of seven configurations of platform incentives leading to high SCA. Among them, the consistency of each configuration is above 0.8, and the overall consistency and overall coverage are 0.8839 and 0.824, respectively, which meets the requirements of the qualitative comparative analysis method for coverage and consistency (42), indicating that the seven configurations have strong explanatory power to the results.

### Robust Test

This article conducts a robust test of the antecedent configurations of high KCP (48). We reset the threshold of the number of cases from 2 to 3, the resulting configurations are the same (Supplementary Tables 1, 2). then, increasing the original consistency threshold from 0.80 to 0.85, the resulting configurations are consistent (Supplementary Tables 3, 4), which show that the results obtained in this study are robust.

## Discussions

Under the background of the COVID-19 pandemic, OKCs have become a place for people to seek knowledge and build virtual social circles. Therefore, to fully mobilize the enthusiasm of users to participate in knowledge collaboration and build a healthier and more sustainable platform ecology, the platform should fully understand the different demands of users, and construct different incentives for different types of users from the perspective of configuration.

For the ICA, platforms relying only on the market or social incentives both can drive the appreciation of intellectual capital, while the effect of the former is relatively poor. At the same time, the differentiated combination of the two types can also achieve the goal, and the role of social incentives in various configurations is symmetric, which is the core condition of existence, while market-oriented incentives are asymmetrical. The 10 configurations can be divided into four types, as follows.

### Market-Oriented Incentives Driven

In configuration 1, virtual currency and cash benefit are both the core existence conditions, while the social identity and skill training are both the peripheral absent conditions to drive the ICA. This configuration shows that only relying on material incentives represented by virtual currency and cash benefits can promote knowledge production. In the era of the knowledge economy, the knowledge payment model prompts more and more users to obtain rewards by providing their heterogeneous knowledge. The users who get paid by answering questions and providing specific knowledge are knowledge contributors, and their participation is crucial to the healthy development of OKC (12). The community should pay full attention to the market-oriented needs of knowledge contributors, and create more convenient communication and knowledge sharing channels for them, such as the paid consultation column in Zhihu. At the same time, platforms should pay attention to the strength of market-oriented incentives to avoid negative effects due to the erosion of economic incentives on knowledge sharing (7).

### Social Incentives Driven

This type of configuration includes four paths. Among them, configuration 2a takes skill training and communication as the core existence conditions, and configurations 2b, 2c, and 2d take social identity, community reputation, and skill training as the core existence conditions, respectively, indicating that social incentives play a very important role in the improvement of ICA, which further verifies the conclusions of Rafaeli et al. (20) and Wolfe and Loraas (10). As a knowledge platform with strong self-organization, the active participation and active contribution of users are very important (12). The users who only pursue spiritual benefits or aim to communicate and improve their skills are often knowledge contributors and have a high willingness to contribute knowledge (12). For such users, the platform can enhance and consolidate their contributions through social incentives such as level promotion, user privileges, setting up topic areas, and selection of outstanding participants to improve ICA.

### Social Incentives Leading

This type of configurations includes two paths, both with social identity, community reputation, and skill training as the core existing conditions, and virtual currency and cash benefit as the peripheral present conditions, respectively. These configurations show that the appreciation of intellectual capital needs to be driven by social incentives, and supplemented by a little material incentive. And when the platform has fewer resources or low strength for market incentives, it can improve user enthusiasm through higher social recognition, community reputation, and social incentives, such as skill training.

### Combined Driven

The fourth type of configuration includes three paths, which all include two market-oriented incentives (virtual currency and cash benefit) and are supplemented by two social incentives to improve ICA. It is worth noting that these configurations do not require incentives for skill improvement. It can be seen that the target users of this type are the groups who hope to obtain certain heterogeneous knowledge through knowledge interaction, and at the same time show their value in the virtual community, and gain respect from others (12). Such users have relatively rich knowledge and skills themselves, and they are important participants in OKC knowledge interaction. In the era of a knowledge economy, the improvement of people's consumption level, the establishment of mobile payment habits, and the change of information dissemination methods have jointly promoted the evolution of knowledge sharing from “free” to paid. On the one hand, knowledge owners use the OKC platform to “realize knowledge.” On the other hand, they provide valuable information and knowledge for many ordinary users, which can increase user stickiness and scale. This interdependent and mutually reinforcing relationship between knowledge contributors and seekers provides a guarantee for a healthy and sustainable platform ecology (4).

Compared with ICA, the configurations for SCA pay more attention to the role of social incentives as a whole, especially the corresponding incentives for community communication.

### Communication Driven

This type of configuration includes two paths, and communication is the core existence condition of them, while the cash benefit and community reputation are the peripheral absent conditions, and skill training is a peripheral existence condition for configuration 1a, and configuration 1b complements the social identity, community reputation, and skill training as the peripheral absent conditions and virtual currency as peripheral existence condition, which can also drive the appreciation of social capital. This type of configuration shows that communication plays a very important role in driving the SCA in OKC. Social capital is the connection between people and the personal wealth accumulated through connection. It is the trust cooperation and collective behavior established in the interpersonal network of the community (29), which itself is the result of interpersonal interaction. The OKC provides a good platform and opportunity for users whose main purpose is to seek interpersonal interaction. For the maintenance of such users, the interactive functions and related experience of the platform are particularly important.

### Cash Benefit Driven Under the Leadership of Social Incentives

This type of configuration includes two paths, both of which are based on cash benefits. Path 2a complements social identity and community communication as core existence conditions, and path 2b complements social identity and skill training as the core existence conditions and community reputation as the peripheral existence condition. These configurations encourage users to participate in knowledge collaboration by enhancing their sense of social identity, personal knowledge, and skills, as well as convenient online interaction and certain cash benefits. Members with heterogeneous knowledge can obtain certain economic benefits by participating in knowledge interaction. At the same time, in the process of improving their skills, showing their value, gaining respect from others, and their sense of achievement are the main reasons for their participation in OKC (12). So, the generation of KCP requires the platform to take into account both social and economic incentives.

### Virtual Currency Driven Under the Leadership of Social Incentives

This type of configuration includes three paths, all of which are based on virtual currency and community reputation as the core existence conditions. Configuration 3a complements communication as the core existence condition and cash benefit as the peripheral existence condition. Configuration 3b takes the cash benefit as the peripheral existence condition, and configuration 3c takes the community reputation, social identity, and skill training as the core existence conditions to develop the SCA. This type of configuration is similar to the second one, which requires the combined effect of social and market-oriented incentives. While the difference is that the configurations of type 3 pay more attention to virtual currency. Virtual currency (such as Zhihu Coin and Live Salt Coin) is generally used for internal circulation in OKC, which purpose is to facilitate users to purchase corresponding services, motivate or reward users for participation and contribution, and can also increase their stickiness. For users who pay attention to social incentives such as community reputation, have certain loyalty and a certain demand for market-oriented incentives, the platform can formulate corresponding incentives according to such contributions.

## Conclusions

### Research Conclusions

This research uses the fsQCA method to analyze the configurations of platform incentives for high KCP in OKC, and analyzes the heterogeneity of the configurations for ICA and SCA. The main research conclusions are as follows: The single factors of virtual currency, cash benefit, social identity, community reputation, skill training, and communication cannot constitute the necessary conditions to drive the improvement of ICA and SCA, and each factor needs to be coordinated to achieve better incentives. There are four types of configurations for ICA and three types of configurations for SCA. There is heterogeneity in the configurations of ICA and SCA, and compared with the former, the latter's incentive configurations as a whole emphasize the role of social incentives, especially the corresponding incentives for communication, and it is difficult to realize the appreciation of social capital only by market-oriented incentives. For the three types of platform incentives for high SCA, social incentives occupy the main position, while it is more efficient when supplemented by certain economic incentives are more effective.

### Managerial and Policy Implications

First of all, the overall performance of the OKC needs to be comprehensively considered, including both ICA such as new knowledge acquired by users and platforms and SCA such as closer connections among users and between users and platforms. The two groups of people (knowledge producers and consumers) and the two types of performance need to promote each other. Therefore, the platform should not only attach importance to the incentives of high-level knowledge producers, but also ordinary users and knowledge seekers.

Second, the research results show that there is a demand–fit relationship between different user groups and incentives configurations. For example, in the configurations of ICA, pure social incentives can stimulate ~20% of the population to actively participate. While the combination of social and market-oriented incentives can increase this ratio to more than 50%. And for the SCA, pure social incentives can also increase this ratio to more than 60%, which also indirectly proves the limitations of market-oriented incentives. Platforms can further optimize the incentive system according to the different psychological needs of users to avoid simple economic stimulation.

Third, while paying attention to the effectiveness of social incentives, the platform must also pay special attention to the existence of “wool-hunting parties.” This research shows that nearly 30% of platform users may participate in content production under pure market-oriented incentives. Combined with previous studies, it is found that the excessive use of market means will lead to the loss of knowledge quality and social interaction, which may cause the Matthew effect and speculative behavior, and change the interaction and collaboration among users. It is necessary for the platform to discover such phenomena and users, and to make timely improvements in terms of platform rules and incentive systems.

Fourth, in the configurations of ICA, social identity and community reputation are the core existence conditions of the five paths; followed by skills training, virtual currency, and cash benefit, which appear four times as the core existence conditions. In terms of promoting knowledge production, the platform needs to pay special attention to the social–psychological needs and the improvement of the abilities of users.

Fifth, for the configurations of SCA, the communication as the core existence condition appears 4 times, followed by virtual currency, social identity, and community reputation, which appear 3 times, respectively. It shows that in terms of promoting the creation of a community atmosphere, the platform should pay attention to social incentives, and at the same time, it should be supplemented by appropriate economic incentives.

Sixth, through the analysis of configurations, the platform users can be subdivided as a whole, and user groups with differentiated needs can be found, which has a certain auxiliary effect on user portraits.

### Limitations and Further Research

Although the configurations of platform incentives proposed in this article provide a certain reference for the efficient and sustainable development of the platform, it also has certain limitations. The specific performance is as follows: First, the platform incentives designed in this study only include two types of six variables. In the future, more complex incentive mechanisms can be considered to enrich the research model. Second, our research is based on cross-sectional data and failed to try to explore the long-term effects of these motivational factors from the time dimension. And our research on OKC's knowledge collaboration mechanism is not deep enough. In future research, we will pay more attention to collecting data from more OKCs, establish a long-term tracking and investigation system, explore the configuration path of the platform incentives for OKC's KCP from a dynamic perspective, and deepen the research of OKC's knowledge collaboration mechanism.

## Data Availability Statement

The original contributions presented in the study are included in the article/Supplementary Material, further inquiries can be directed to the corresponding author.

## Ethics Statement

Ethical review and approval was not required for the study on human participants in accordance with the local legislation and institutional requirements. Written informed consent for participation was not required for this study in accordance with the national legislation and the institutional requirements.

## Author Contributions

JC conceived and designed the study and designed the model. BC collected the parameters. YZ did the data analyses and contributed to the writing of the manuscript. All authors interpreted the results and approved the final version for publication.

## Funding

This work was supported by the Beijing Municipal Natural Science Foundation (9222012) and the National Natural Science Fund Project (71572015). The funder of the study had no role in the design of the study and collection, analysis, and interpretation of data, and in the writing the manuscript.

## Conflict of Interest

The authors declare that the research was conducted in the absence of any commercial or financial relationships that could be construed as a potential conflict of interest.

## Publisher's Note

All claims expressed in this article are solely those of the authors and do not necessarily represent those of their affiliated organizations, or those of the publisher, the editors and the reviewers. Any product that may be evaluated in this article, or claim that may be made by its manufacturer, is not guaranteed or endorsed by the publisher.
