# Supplementary material for: The Configuration of Incentives in Small and Medium-Sized Content Platform Enterprises Under the Normalization of COVID-19
Source: Front Public Health. 2022 Apr 29;10:885729. doi: 10.3389/fpubh.2022.885729 (PMC9099008; doi:10.3389/fpubh.2022.885729)
Supplement: Supplementary file 1 [file Data_Sheet_1.docx]

Supplementary Material

# Supplementary Tables

Robustness Test Results

Tab 1. The configurations leading to high ICA

| conditions | configurations | | | | | | | | | | |  |
| --- | --- | --- | --- | --- | --- | --- | --- | --- | --- | --- | --- | --- |
|  | 1 | 2a | 2b | 2c | | 2d | 3a | 3b | 4a | 4b | 4c |  |
| Virtual currency | ● |  |  |  | |  |  | ● | ● | ● | ● |  |
| Cash benefit | ● |  |  |  | |  | ● |  | ● | ● | ● |  |
| Social identity |  |  |  |  | | ● | ● | ● | ● | ● |  |  |
| Community reputation |  |  |  |  | | ● | ● | ● | ● |  | ● |  |
| Skill training |  | ● | ● | ● | |  | ● | ● |  | ● |  |  |
| Communication |  |  | ● | ● | |  |  | ● |  | ● | ● |  |
| Consistency | 0.8701 | 0.9336 | 0.9708 | 0.9700 | | 0.9265 | 0.9527 | 0.9846 | 0.9301 | 0.9832 | 0.9597 |  |
| Raw coverage | 0.2496 | 0.2636 | 0.2715 | 0.2641 | | 0.2436 | 0.2785 | 0.5275 | 0.3423 | 0.5033 | 0.5342 |  |
| Unique coverage | 0.0085 | 0.0227 | 0.0045 | 0.0061 | | 0.0120 | 0.0091 | 0.0323 | 0.0062 | 0.0120 | 0.0245 |  |
| Overall solution consistence | | | | | 0.9090 | | | | | | |  |
| Overall solution coverage | | | | | 0.7256 | | | | | | |  |

notes: Black circles (●) indicate the presence of a condition, and circles with x () indicate its absence. large circles mean core condition, small circles mean peripheral condition. Black spaces mean “does not care” condition.

Tab 2. The configurations leading to high SCA

| conditions | configurations | | | | | | |
| --- | --- | --- | --- | --- | --- | --- | --- |
|  | 1a | 1b | 2a | 2b | 3a | 3b | 3c |
| Virtual currency |  |  |  | ● | ● | ● | ● |
| Cash benefit |  |  | ● | ● | ● | ● |  |
| Social identity |  |  | ● | ● |  | ● | ● |
| Community reputation |  |  | ● | ● | ● |  | ● |
| Skill training | ● | ● | ● |  |  | ● | ● |
| Communication | ● | ● |  |  | ● | ● | ● |
| Consistency | 0.9539 | 0.9448 | 0.9419 | 0.9103 | 0.9405 | 0.9656 | 0.9628 |
| Raw coverage | 0.3254 | 0.3138 | 0.3358 | 0.4086 | 0.6386 | 0.6029 | 0.6293 |
| Unique coverage | 0.0055 | 0.0053 | 0.0123 | 0.0088 | 0.0253 | 0.0154 | 0.0378 |
| Overall solution consistence | | | 0.9053 | | | | |
| Overall solution coverage | | | 0.7935 | | | | |

notes: Black circles (●) indicate the presence of a condition, and circles with x () indicate its absence. large circles mean core condition, small circles mean peripheral condition. Black spaces mean “does not care” condition.

Tab 3. The configurations leading to high ICA

| conditions | configurations | | | | | | | | | | |  |
| --- | --- | --- | --- | --- | --- | --- | --- | --- | --- | --- | --- | --- |
|  | 1 | 2a | 2b | 2c | | 2d | 3a | 3b | 4a | 4b | 4c |  |
| Virtual currency | ● |  |  |  | |  | ● |  | ● | ● | ● |  |
| Cash benefit | ● |  |  |  | |  |  | ● | ● | ● | ● |  |
| Social identity |  |  | ● |  | |  | ● | ● | ● |  | ● |  |
| Community reputation |  |  |  | ● | |  | ● | ● |  | ● | ● |  |
| Skill training |  | ● |  |  | | ● | ● | ● |  |  |  |  |
| Communication |  | ● |  |  | |  |  |  | ● | ● |  |  |
| Consistency | 0.8680 | 0.9741 | 0.9013 | 0.8804 | | 0.9336 | 0.9726 | 0.9638 | 0.9606 | 0.9597 | 0.9441 |  |
| Raw coverage | 0.2986 | 0.3075 | 0.2633 | 0.2534 | | 0.2636 | 0.5459 | 0.5216 | 0.5386 | 0.5342 | 0.5447 |  |
| Unique coverage | 0.0049 | 0.0171 | 0.0053 | 0.0056 | | 0.0151 | 0.0366 | 0.0120 | 0.0157 | 0.0095 | 0.0044 |  |
| Overall solution consistence | | | | | 0.8856 | | | | | | |  |
| Overall solution coverage | | | | | 0.7592 | | | | | | |  |

notes: Black circles (●) indicate the presence of a condition, and circles with x () indicate its absence. large circles mean core condition, small circles mean peripheral condition. Black spaces mean “does not care” condition.

Tab 4. The configurations leading to high SCA

| conditions | configurations | | | | | | |
| --- | --- | --- | --- | --- | --- | --- | --- |
|  | 1a | 1b | 2a | 2b | 3a | 3b | 3c |
| Virtual currency |  | ● |  | ● | ● | ● | ● |
| Cash benefit |  |  | ● | ● | ● |  | ● |
| Social identity |  |  | ● | ● |  | ● |  |
| Community reputation |  |  | ● |  | ● | ● | ● |
| Skill training | ● |  | ● |  |  | ● |  |
| Communication | ● | ● |  | ● |  |  | ● |
| Consistency | 0.9481 | 0.9337 | 0.9388 | 0.9481 | 0.8922 | 0.9422 | 0.9405 |
| Raw coverage | 0.3650 | 0.3178 | 0.6197 | 0.6485 | 0.4249 | 0.6450 | 0.6386 |
| Unique coverage | 0.0381 | 0.0027 | 0.0158 | 0.0204 | 0.0136 | 0.0433 | 0.0081 |
| Overall solution consistence | | | 0.8240 | | | | |
| Overall solution coverage | | | 0.8839 | | | | |

notes: Black circles (●) indicate the presence of a condition, and circles with x () indicate its absence. large circles mean core condition, small circles mean peripheral condition. Black spaces mean “does not care” condition.
